# Post-exposure effects of simulator sickness in motion-based flight simulators: a review of symptom duration and implications for flight fitness

**DOI:** 10.3389/fphys.2026.1848400

**Published:** 2026-09-21

**Authors:** Nico Willebrand genannt Overgahr, David Feibus, Stefan Sammito

**Affiliations:** 1 33^rd^ Tactical Air Wing, Büchel Air Base, Büchel, Germany; 2 Occupational Medicine, Faculty of Medicine, Otto-von-Guericke-University of Magdeburg, Magdeburg, Germany; 3 German Air Force Centre of Aerospace Medicine, Group Research & Development, Cologne, Germany; 4 Engineer and Scienctist Exchange Program, United States Air Force, Cologne, Germany

**Keywords:** aerospace medicine, flight safety, flight simulator, full-motion simulator, military aviation, motion sickness, simulator sickness

## Abstract

**Introduction:**

The increasing use of modern motion-based flight simulators in military and civil aviation, despite their high relevance for training, is accompanied by the occurrence of simulator sickness. While acute symptoms during simulation are well described, the duration of post-exposure complaints and their significance for subsequent real flight capability have not yet been sufficiently investigated. The aim of this exploratory review was to summarise the available studies on the (persistence of) symptoms after flight-related simulator training.

**Methods:**

A systematic literature search was conducted in PubMed, Web of Science and Scopus in accordance with PRISMA guidelines. From a total of 4,404 hits, 14 full texts were included in this review.

**Results:**

The studies showed considerable heterogeneity in terms of participants, simulator types, exposure duration and measuring instruments. Typical symptoms included eye strain, nausea, disorientation and, less frequently, dizziness and fatigue. Only a few studies systematically investigated after-effects beyond the immediate period following the simulation. The limited available data suggest that most complaints subside significantly within four to six hours, although individual cognitive and postural impairments may persist for longer. It is currently not possible to make reliable statements about the objective restoration of flight fitness or an evidence-based minimum waiting period between simulator training and actual flight.

**Discussion:**

Overall, there is a considerable need for research, particularly using modern full-motion simulators and standardised objective performance parameters, for deriving valid safety-related recommendations and examining symptom decay.

**Systematic Review Registration:**

https://www.crd.york.ac.uk/PROSPERO/, identifier CRD420251161357

## 1 Introduction

The use of sophisticated flight simulators, especially those with moving platforms, has increased significantly in military and civil aviation in recent decades (Balcerzak and Kostur, 2018). The use of such systems enables efficient and cost-effective training, as virtually any conceivable environment can be realistically simulated and complex scenarios can be practised under controlled conditions without risk and independently of the environment. Technological advances in 3D motion-based simulators in particular have made it possible to achieve almost realistic flight operations. This not only reduces the high costs of real flight hours (Callender, 2008), but also makes the training processes more efficient overall.

Despite optimised training and realistic representation, undesirable side effects occur over time, which are summarised under the term “simulator sickness”. Simulator sickness describes a form of motion and/or visual induced motion sickness that can occur during and after the use of simulators. Motion sickness is caused by sensory discrepancies between visual, vestibular and proprioceptive information.; visually induced motion sickness is diagnosed when the stimulus is visual motion (Cha et al., 2021). It is caused by sensory discrepancies between visual, vestibular and proprioceptive information. While the eyes perceive movement, the vestibular system registers no acceleration or an incongruous acceleration. This sensory imbalance leads to faulty processing in the central nervous system, which causes the typical symptoms (Benson, 2002). The most common symptoms include nausea, dizziness, paleness, headaches, cold sweats and light-headedness. Both visual symptoms, such as eye strain, and cognitive impairments, such as disorientation, have been reported (Kennedy et al., 1989; Crowley, 1987; Muth, 2009).

Even after leaving the simulator, pilots may experience symptoms such as balance disorders, spatial disorientation and prolonged reaction times (Ungs, 1989). With such symptoms, subsequent real flight training after simulator flight is associated with risks and jeopardises flight safety. Therefore, in practice, it is often recommended to take a break after simulator training before flying again (Kommando Heer, 2020). However, there are no scientific recommendations specifying a specific waiting period. For example, the US Federal Aviation Administration (FAA) merely points out that pilots are not allowed to fly if they experience symptoms of illness or disorientation (Federal Aviation Administration (FAA), 2024).

The aim of this exploratory review article is therefore to (1) summarise, based on existing scientific literature, the after-effects of simulator training/use and (2) if possible, establish the duration of their persistence after simulator training, and whether there are studies on when pilots are safe to fly again following simulator training. These findings could serve as a basis for future guidelines and further increase safety in flight operations.

## 2 Materials and methods

The literature review was based on a systematic search using the PRISMA criteria (Page et al., 2021) in the PubMed, Web of Science and Scopus databases. On 25 November 2025, predefined search parameters “((Motion sickness) OR (Simulator Sickness) OR (Cinerama Sickness) OR (Cyber Sickness)) AND ((Postural Instability) OR (Postural Stability) OR (Mitigation) OR (Symptoms))” were used to identify relevant scientific publications. An overview of the methodological approach is presented in **Figure 1**. The search was registered in the PROPERO database (CRD420251161357).

**Figure 1:**
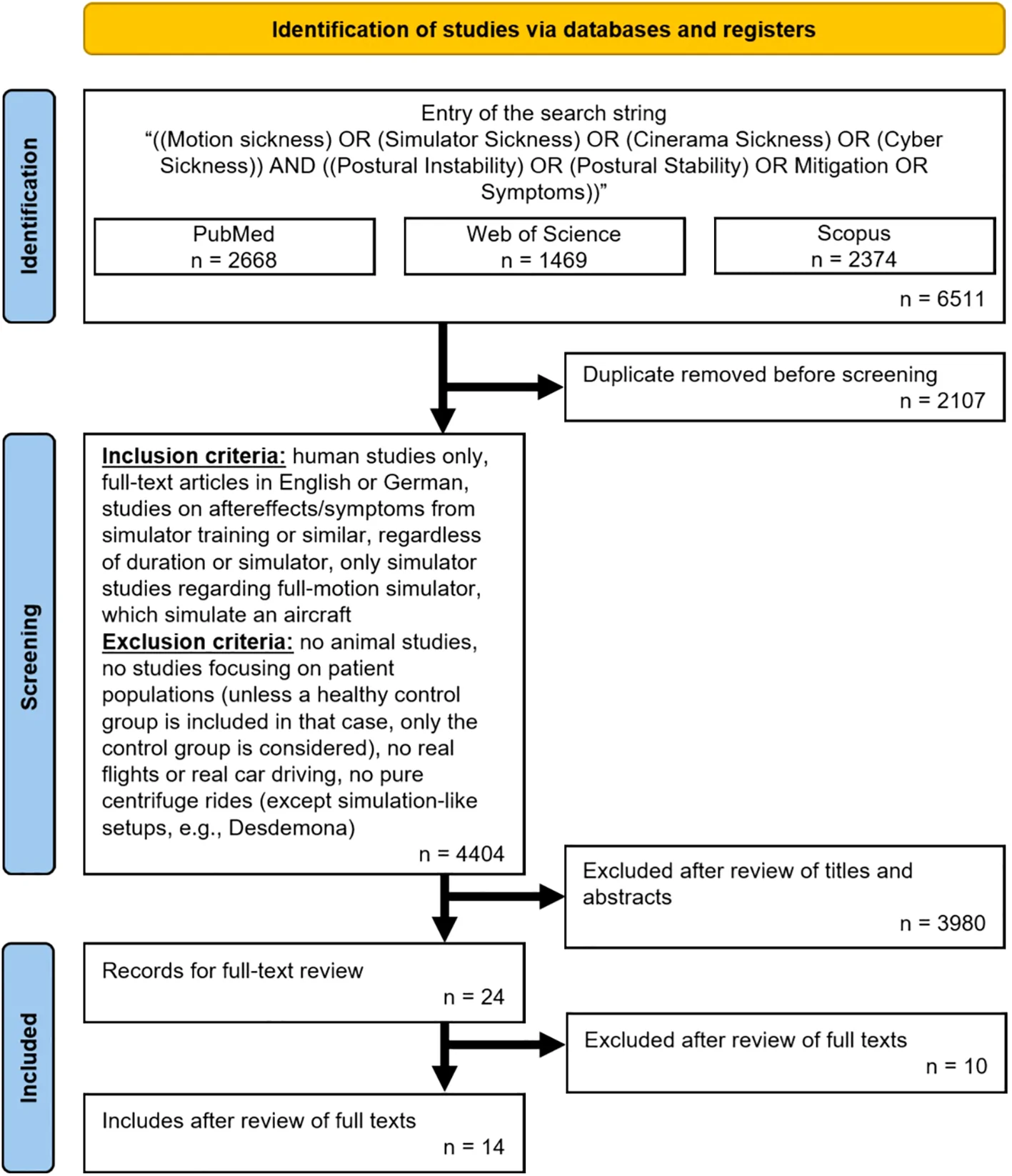
PRISMA flowchart for literature searches.

After removing duplicates from the search results, a total of 4,404 hits remained, which were initially evaluated by reviewing the titles and abstracts by two persons. The inclusion criteria for subsequent analysis in this exploratory review were studies involving human subjects that were published in English or German. We have included German studies as a matter of principle, as the research team was able to read them in the original language and because the databases, that were used in this search, also list German journals with English titles, abstracts and keywords. This was done to ensure that potential studies were not excluded due to a language restriction limiting the search to English-language journals only. The studies should have investigated the after-effects or symptoms associated with simulator training in the field of aviation or comparable exposures, regardless of the duration of training. Only studies that used a simulation scenario depicting the piloting or flying in an aircraft were considered.

Animal studies and studies focusing exclusively on groups of patients with pre-existing conditions were not included. However, if such studies included a healthy control group, only this group was included in this exploratory review. Studies involving actual flights were also excluded, as were studies in which the participants were exposed to increased G-forces exclusively in a human centrifuge but did not include any simulation elements. If simulation elements were the main focus and these were carried out in a human centrifuge, they were included in the analysis. **Table 1** shows an overview regarding the population, intervention, comparison and outcome (PICO) of our review.

**Table 1:** PICO-overview.

PICO-Component	Content
Population	Human in a simulator scenario as a pilot or flying in an aircraft
Intervention	Simulator flight scenario
Comparison	Effects (symptoms) after versus before before the simulator flight
Outcome	When are symptoms back on the level before simulator flight

After the titles and abstracts were reviewed independently by two reviewers (NW and DF), a total of 3,980 articles had to be excluded, leaving 24 articles for further evaluation of the full text. Of these, ten articles were excluded after a full-text search because they focused primarily on technical developments of the simulators without investigating the effects or after-effects on the human body. After reviewing the full texts, a total of 14 full texts remained, which were included in this exploratory review (Kennedy et al., 1989; Crowley, 1987; Muth, 2009; Ishak et al., 2018; Kaufeld and Alexander, 2019; Kim et al., 2023; Webb et al., 2009; Adelstein et al., 2022; Sharkey and McCautley, 1992; Kennedy et al., 1987; Ben-Ari et al., 2023; Nakdimon et al., 2025; Blok, 1992; Javaid et al., 2024). A complete overview of the results and the studies listed can be found in **Table 2**.

**Table 2:** Overview over the included studies.

	Number of participants	Age [years]	Test group (for pilots: average flight experience)	Simulator	Tasks for the test participants	Duration of the simulation	Simulator sickness measurement methods	Frequency of tests	Duration of symptoms	Symptoms after simulator flight
Kennedy et al., 1989	1186 “flights”	n/a	pilots (n/a)	6-DOF and no-Motion	Active flight without specifications	n/a	Before and after the flight: MSSC, standing on one leg, walking on a line. One-time MHQ.	Before and after the flight	n/a	Significant increase in nausea, fatigue and eye strain, rarely vomiting or retching
Sharkey and McCautley, 1992	10 m	26.3	Pilots (601.4 hours, including 534.6 hours on rotary wing aircraft and 80.9 hours in the simulator)	6-DOF (NASA Vertical Motion Simulator [VMS])	Active flight with specifications	approx. 1 hour	SSEQ, SOLEC. WOFEC, 7-point well-being scale	Before, immediately after and 30 minutes after simulator flight. Well-being checked every 60 seconds during the flight.	Partially over the 30-minute observation period	Increased nausea, disorientation, eye strain, postural instability
Webb et al., 2009 * control group only	12 m, 5 f	29 (19-42)	general civilian population”	Multi Axis Ride Simulator (MARS)	Passive flight as a passenger	3 x 20 minutes on three days	Before simulation: MHQ. After simulation: mod. SSQ with a point scale of 0-5, postural balance test and PVT	Before and after each run	n/a	Increased tiredness, drowsiness and eye strain
Adelstein et al., 2022	19 m, 4 f	42.8 (20-67)	Civilians without flying experience	6-DOF (NASA Vertical Motion Simulator [VMS])	Passive flight as a passenger	10 minutes on two days	5 min PVT, SSQ and NASA TLX, one-time MSSQ, 2-channel ECG, heart and respiratory rate, skin temperature, electrodermal activity	PVT before the flight and once during the flight, SSQ and NASA TLX immediately afterwards	n/a	Elevated SSQ scores
Ben-Ari et al., 2023 * control group only	40 m,1 f	30.5 ± 7.1	Pilots (n/a)	3-DOF (IAF Helicopter Aircraft Weapon System Trainer)	Active flight without specifications	75 minutes	SSQ	0 min baseline SSQ, then 45 min debriefing and start of exercise, after 120 min 2nd SSQ, then second exercise, after 210 min 3rd SSQ, then third exercise, and after 300 min 4th SSQ	Up to 300 minutes after simulation	Lower values for the SSQ (placebo)
Blok, 1992	141	n/a	Pilots (n/a)	6-DOF (UH-60A 2B38 BlackHawk flight simulator)	Active flight without specifications	n/a	Questionnaire	One-off after simulator flight	No longer than 6 hours	Increased nausea, dizziness, sweating, headaches
Crowley, 1987	115 (112 complete questionnaires)	n/a	Pilots (n/a)	6-DOF (AH-1 Cobra 2B33 Flight Weapons Simulator [FWS])	Active flight without specifications	n/a	mod. questionnaire, see symptoms	One-off after simulator flight	n/a	Increased nausea, sweating, dizziness, headaches, vomiting, illusion of movement, eye strain and fatigue
Ishak et al., 2018	4 m, 7 f	27.7 (19-50)	no pilots	3-DOF (InMotion Flight Simulator)	Passive flight as a passenger	2 x 10 minutes	SSQ	before and after each simulation	n/a	Significantly increased SSQ values under visual conditions (including nausea, disorientation, and eye strain), significant reduction under occlusive conditions
Javaid et al., 2024	2 m, 3 f	24 (22-27)	no pilots	2-DOF (self-developed)	Passive flight as a passenger	n/a	SSQ	One-off after simulation	n/a	Elevated SSQ scores (nausea, disorientation, and eye strain): roll > pitch condition
Kaufeld and Alexander, 2019	28 m, 2 f	19.3	no pilots	6-DOF (Brunner, HMD Oculus Rift CV1)	Passive flight as a passenger	2x30 minutes	SSQ, MSSQ (eye test)	Before, immediately after the simulation and 1 hour after the end of the simulation	1 hour	Elevated SSQ scores (nausea, disorientation, and eye strain)
Kennedy et al., 1987	191	n/a	Pilots and flight students (n/a)	6-DOF (2F64C helicopter simulator SH-3 Sea King) and 6-DOF (Motion Base 2F87F fixed-wing simulator P-3C Orion)	Active flight without specifications	n/a	MSQ, WOFEC, SONLEC	Before, during and after the flight MSQ; WOFEC and SONLEC only after the flight	n/a	High MSQ values in the helicopter simulator, practically no increase in MSQ values in the aeroplane simulator, no impairment of balance in either case.
Kim et al., 2023	36	26.1 (24-30)	no pilots	6-DOF (Sperical Motion Platform [SMP])	Active flight with specifications	40 minutes	MSSQ, SSQ, physiological measurements with Empatica E4 wristband, SS	SSQ immediately after simulation, physiological data continuously during flight	n/a	Elevated SSQ values (nausea, disorientation, and eye strain): no motion > motion. Increased skin conductance: no motion > motion. Elevated heart rate; increased pulse volume in motion group; skin temperature not usable.
Muth, 2009	11 m	32	Pilots (600 hours)	2-DOF	Active flight with specifications	Maximum one hour	MSHQ, MSAQ, Cognitive test battery, sharpened Romberg Test, DVAT	Before exposure, immediately after (0 h) and after 2, 4, 6, 8 and 24 h; Every 10 minutes during the simulation MSAQ	After-effects lasted between 2 and 4 hour postexposure	Significant cognitive impairment, balance also impaired, MSAQ with only slight increase, no change in dynamic visual acuity
Nakdimon et al., 2025	207	n/a	Pilots (n/a)	6-DOF (IAF Helicopter Aircraft Weapon System Trainer)	Active flight without specifications	n/a	SSQ	One-off after the last simulator flight	n/a	Increased SSQ values in over 50% of pilots

DOF, Degree of Freedom; DVAT, Dynamic Visual Acuity Test; f, female; m, male; MHQ, Motion History Questionnaire; MMSC, Motion Sickness Symptom Checklist; MSAQ, Motion Sickness Assessment Questionnaire; MSHQ, Motion Sickness History Questionnaire; MSQ, Motion Sickness Questionnaire ; n/a, no available; NASA TLX, NASA Task Load Index; PVT, Psychometric Vigilance Test; SOLEC, Stand-on-leg eyes closed; SONLEC, Standing On Non-Preferred Leg Eyes Closed; SSEQ, Simulator Side Effects Questionnaire; SS, Simulator Sickness; SSQ, Simulator Sickness Questionnaire; WOFEC, Walking-Toe-to-Heel-Eyes Closed

## 3 Results

From the 14 included studies in this exploratory review nine studies have only examined the after-effects directly after the simulator session and only five studies have examined after-effects of simulator training/use over time with measurement and/or with questionaires. The studies are first presented in their entirety below to illustrate the side effects of simulator training; with regard to the duration of these effects, however, only the five studies that explicitly examined them over time will be analysed in the following sections. All included studies dealt with different groups of people, types of simulators, test methods and symptoms in order to systematically record the onset, intensity and duration of motion sickness.

### 3.1 Participation sizes

Most studies examined only small groups of participants with fewer than 100 participants (Muth, 2009; Ishak et al., 2018; Kaufeld and Alexander, 2019; Kim et al., 2023; Webb et al., 2009; Adelstein et al., 2022; Sharkey and McCautley, 1992; Ben-Ari et al., 2023; Javaid et al., 2024). Only three studies had groups of more than 100 participants (Crowley, 1987; Kennedy et al., 1987; Blok, 1992), and one study recorded 1,186 “flights” (Benson, 2002), although a pilot could have performed several flights. The average age of the participants ranged from 19.3 to 42.8 years, with five studies (Benson, 2002; Crowley, 1987; Kennedy et al., 1987; Nakdimon et al., 2025; Blok, 1992) providing no information on the age of the participants. The studies included both pilots with extensive flying experience (in some cases several hundred actual flying hours) and civilian participants with no flying experience whatsoever. In eight of the 14 studies (Kennedy et al., 1989; Crowley, 1987; Muth, 2009; Sharkey and McCautley, 1992; Kennedy et al., 1987; Ben-Ari et al., 2023; Nakdimon et al., 2025; Blok, 1992), only pilots were included as participants in the study, while the other six studies (Ishak et al., 2018; Adelstein et al., 2022; Javaid et al., 2024) examined non-pilots in order to analyse reactions to simulated movement.

### 3.2 Simulator type

The simulators were comprised of systems with two to six degrees of freedom (DOF) on various complex motion platforms. Depending on the experimental design, the scenarios varied between active flights, in which the participants themselves controlled the aircraft (in nine studies (Kennedy et al., 1989; Crowley, 1987; Muth, 2009; Kim et al., 2023; Sharkey and McCautley, 1992; Kennedy et al., 1987; Ben-Ari et al., 2023; Nakdimon et al., 2025; Blok, 1992)) and passive flights, in which they acted merely as observers or passengers (in 5 studies (Ishak et al., 2018; Kaufeld and Alexander, 2019; Webb et al., 2009; Adelstein et al., 2022; Javaid et al., 2024)). The duration of the simulations ranged from short 10-minute sessions (Ishak et al., 2018; Adelstein et al., 2022) to simulations lasting 75 minutes (Ben-Ari et al., 2023). Six of the published studies did not provide any information on the duration of the simulations (Kennedy et al., 1989; Crowley, 1987; Kennedy et al., 1987; Nakdimon et al., 2025; Blok, 1992; Javaid et al., 2024). In two studies (Webb et al., 2009; Adelstein et al., 2022), repeated simulations were carried out over several days, and in two studies (Ishak et al., 2018; Kaufeld and Alexander, 2019), the simulation exposure was carried out twice over an identical duration.

### 3.3 Publicaton age

The publication age of the included studies varied greatly. Five of the included studies were published before 2000 (Kennedy et al., 1989; Crowley, 1987; Sharkey and McCautley, 1992; Kennedy et al., 1987; Blok, 1992), two studies between 2000 and 2009 (Muth, 2009; Webb et al., 2009), two studies between 2010 and 2019 (Ishak et al., 2018; Kaufeld and Alexander, 2019) and five studies since 2020 (Kim et al., 2023; Adelstein et al., 2022; Ben-Ari et al., 2023; Javaid et al., 2024; Nakdimon et al., 2025). This is particularly important for the assessment of the underlying simulator technology and transferability to newer simulators.

### 3.4 Symptoms

In terms of symptoms, typical patterns were observed in almost all studies (Kennedy et al., 1989; Crowley, 1987; Muth, 2009; Ishak et al., 2018; Kaufeld and Alexander, 2019; Kim et al., 2023; Webb et al., 2009; Adelstein et al., 2022; Sharkey and McCautley, 1992; Kennedy et al., 1987; Nakdimon et al., 2025; Blok, 1992; Javaid et al., 2024). The most common symptoms were eye strain (in 71% of studies), nausea (50%), disorientation (50%), dizziness (14%) and fatigue (14%). Other symptoms included sweating (28%), headaches (14%), balance disorders (7%), motion illusions (7%), light-headedness (7%) and, in rare cases, vomiting or gagging (7%). Based on the five studies with longer follow-up periods, the symptoms lasted for 4–6 hours (Muth, 2009; Sharkey and McCautley, 1992; Ben-Ari et al., 2023; Blok, 1992).

Despite general agreement on the basic trend, the studies also showed some differentiated results. Kennedy et al. (1987), for example, found that helicopter simulators caused significantly stronger symptoms than flight simulators for fixed-wing aircraft. Sharkey and McCautley (1992) observed a significant increase in nausea, disorientation and eye strain during one-hour simulations in the NASA Vertical Motion Simulator, some of which persisted for 30 minutes after the simulation. Webb et al. (2009) reported that fatigue and dizziness in particular increased, while severe symptoms such as vomiting were rare. The study by Adelstein et al. (2022) showed a significant increase in SSQ and NASA-TLX values after the simulation, accompanied by measurable physiological changes. Ben-Ari et al. (2023), on the other hand, found even lower SSQ scores in a control group exposed to a placebo condition, indicating psychological and expectation effects. Ishak et al. (2018) were able to show that visual conditions have a significant influence on the severity of symptoms: Under visual occlusion, SSQ values were significantly lower than under full visual input. Kim et al. (2023) also observed that the so-called no-motion condition caused stronger symptoms and higher SSQ values than the actual movement of the simulator, accompanied by increased skin conductance and heart rate. Muth (2009) found that despite only slight increases in subjectively reported symptoms, cognitive performance and balance were significantly impaired. Finally, a large-scale study by Nakdimon et al. (2025) showed that over 50% of the pilots surveyed had elevated SSQ scores, illustrating the high prevalence of the phenomenon even among experienced flight personnel.

### 3.5 Measurement methods and time of measurement

The measurement methods used to record simulation sickness were varied, with the Simulator Sickness Questionnaire (SSQ) being used as the main instrument in eight studies (Ishak et al., 2018; Kaufeld and Alexander, 2019; Kim et al., 2023; Webb et al., 2009; Adelstein et al., 2022; Ben-Ari et al., 2023; Nakdimon et al., 2025; Javaid et al., 2024). In two studies, physiological parameters were recorded (Kim et al., 2023; Adelstein et al., 2022). In six studies (Kennedy et al., 1989; Muth, 2009; Webb et al., 2009; Sharkey and McCautley, 1992; Kennedy et al., 1987; Adelstein et al., 2022), cognitive and motor performance measures (including postural instability) were used. In addition, the NASA Task Load Index (NASA TLX (Adelstein et al., 2022)) was used to subjectively assess stress levels.

The timing of measurements also varied across studies: In most cases (5 out of 14 studies), symptoms were measured before and after the simulation (Muth, 2009; Kaufeld and Alexander, 2019; Sharkey and McCautley, 1992; Ben-Ari et al., 2023; Blok, 1992), in five studies also several times during the simulation (Muth, 2009; Kim et al., 2023; Adelstein et al., 2022; Sharkey and McCautley, 1992; Kennedy et al., 1987) and/or at intervals after the simulation up to 24 hours (Muth, 2009; Kaufeld and Alexander, 2019; Ben-Ari et al., 2023).

However, only five of the included studies examined after-effects beyond the direct effects after the end of the simulation (Ben-Ari et al., 2023; Blok, 1992; Muth, 2009; Kaufeld and Alexander, 2019; Sharkey and McCautley, 1992). Two of these studies have shown that participants experienced after-effects for less than one hour (Kaufeld and Alexander, 2019; Sharkey and McCautley, 1992), while three other studies have shown that the effects last for 2–4 hours (Muth, 2009) and up to six hours (Ben-Ari et al., 2023; Blok, 1992). Overall, symptoms appear to persist for several hours after simulator training, but mostly disappear after four to six hours. Because of the low number of studies this allows only limited conclusions about the after-effects of simulation sickness, primarily for the acute effects and, based on a really few studies, for longer-term after-effects.

## 4 Discussion

The key finding of this exploratory review is that there are only a very small number of studies (only five) available that explicitly address the after-effects of simulator training in the field of aircraft simulators over time. Although simulator sickness has been known for several decades, many studies have focused primarily on the onset of symptoms during simulation, while post-exposure effects have been investigated much less frequently. In particular, the duration of symptoms after completion of simulator training has hardly been systematically researched to date and is based only on a few studies with limited follow-up periods. However, this is of great importance for operational use, especially in military aviation.

Overall, based on the very limited identified number of studies, symptoms appear to persist for several hours after simulator training, but mostly disappear after 4–6 hours. The symptoms are mostly directly or indirectly associated with the strain: eye strain, nausea, disorientation, dizziness and fatigue.

It should be noted that some of the studies included were published before 2000. These studies are based on simulators whose technical equipment, visual representation and movement algorithms are only comparable to today’s high-performance 3D simulators to a limited extent. The technological development of flight simulators is an important aspect in the interpretation of the results. Modern simulators have high-resolution visual systems, more realistic motion platforms and improved synchronisation between visual and vestibular stimuli. These advances could theoretically contribute to a reduction in sensory discrepancies and thus to a lower incidence of simulator sickness. The transferability of the results of older studies to modern simulator systems is therefore only possible to a limited extent and must be viewed critically. However, it must also be borne in mind that newer 3D simulation environments or virtual reality elements can, in turn, increase the risk of simulator sickness or visually induced motion sickness by causing sensory conflict. So, the results have to been interpreted taking into account the relevant simulator technology used in the studies.

What constitutes a limitation of the exploratory review is the restriction to English- and German-language original studies. This issue must be taken into consideration when evaluating this study. However, it must also be taken into account that the major scientific journals publish in the English language, so ruling out other languages rather meant ruling out national journals. It cannot be excluded that this may have resulted in individual studies not being included in this study, but the authors estimate the resulting bias to be low.

Another limitation is the fact that only a descriptive analysis was possible due to the small number of suitable studies and the marked differences in study design, test participant groups, simulator types, measuring instruments and survey times. The results are therefore based predominantly on a qualitative evaluation. Due to the wide variation in the results, further analyses, such as a meta-analysis, were not possible. Furthermore, the comparability of the studies is limited because both subjective questionnaires and objective performance measures were used inconsistently. Last but not least, several studies lacked important information such as the simulation duration or technical characteristics of the simulators, which makes interpretation and comparability difficult.

A further limitation of the included studies is that the methods used are associated with the symptoms described (primarily eye strain, nausea and disorientation); consequently, there is a certain degree of selection bias in the identification of symptoms due to the measurement methods employed. It is not possible to answer with certainty whether symptoms beyond these were not recorded because the measurement methodology did not provide for them *a priori*, based on the studies described.

The results of this exploratory review highlight a considerable need for further research. Future studies should systematically investigate how long symptoms of simulator sickness persist after completion of training and at what point safe fitness to fly is restored. This requires studies with standardised follow-up periods that record both subjective symptom reports and objective cognitive and motor performance parameters.

In addition, modern simulators with up-to-date visual and full-motion systems should be specifically examined to ensure that the results can be transferred to today’s training conditions. The uniform use of validated measuring instruments and a clear definition of clinically relevant endpoints would significantly improve the comparability of future work. In the long term, the findings could serve as a basis for evidence-based recommendations on the minimum waiting period between simulator training and actual flight, thereby making an important contribution to increasing flight safety.

## Data Availability

The original contributions presented in the study are included in the article/supplementary material. Further inquiries can be directed to the corresponding author.
